# 1208. Omadacycline *In Vitro* Activity Against *Bacillus Anthracis*

**DOI:** 10.1093/ofid/ofab466.1400

**Published:** 2021-12-04

**Authors:** Alisa W Serio, H Carl Gelhaus, Noah Eichelberger, Henry S Heine, Diane M Anastasiou, Kristina Eichhorst

**Affiliations:** 1 Paratek Pharmaceuticals, Inc., King of Prussia, Pennsylvania; 2 MRIGlobal, Kansas City, Missouri; 3 University of Florida, Orlando, Florida

## Abstract

**Background:**

*Bacillus anthracis*, the etiological agent of anthrax, is one of the agents most likely to be used in a biologic attack. Omadacycline previously has demonstrated potent *in vitro *and *in vivo *activity against *B. anthracis*. This project evaluated the *in vitro* activity of omadacycline against a larger set of *B. anthracis* strains across two laboratories.

**Methods:**

Methods: Antibiotic susceptibility testing followed Clinical Laboratory Standard Institute methods against a collection of 53 *B. anthracis* strains at the University of Florida (UF) and 50 *B. anthracis* strains at MRIGlobal, representing human and animal isolates from North America, Africa, Europe, Asia, and Australia. Minimum inhibitory concentrations (MICs) for omadacycline and comparators at both sites (doxycycline, ciprofloxacin, levofloxacin, moxifloxacin) were determined by broth microdilution.

**Results:**

Results: In the UF study, omadacycline demonstrated an MIC50 of 0.015 mg/L and an MIC90 of 0.03 mg/L against *B. anthracis*. Omadacycline MIC values were equal to or lower than doxycycline. In the MRIGlobal study, omadacycline demonstrated an MIC50 of 0.06 mg/L and an MIC90 of 0.06 mg/L (Table 1). All comparator MIC values were within ranges previously observed against these strains. Against a ciprofloxacin-resistant strain (MIC = 2 mg/L), omadacycline had an MIC value of 0.015 mg/L; against a doxycycline-resistant strain (MIC = 4 mg/L), omadacycline had an MIC value of 0.06 mg/L. Reproducibility was observed between the 2 laboratories for omadacycline *in vitro* activity against *B. anthracis* (Table 2).

Table 1. MIC Concentration Summary for Omadacycline and Comparators Against B. anthracis Strains

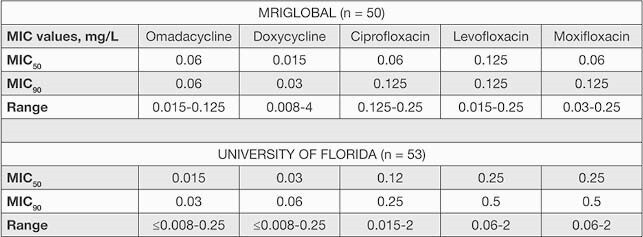

Table 2. Reproducibility of Omadacycline in Vitro Activity Against B. anthracis Strains

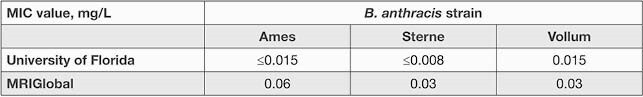

**Conclusion:**

Based on the *in vitro* activity in both studies, omadacycline has the potential to be effective in treating anthrax infection. Reproducibility of omadacycline *in vitro* activity against *B. anthracis* was observed at 2 independent study sites.

**Disclosures:**

**Alisa W. Serio, PhD**, **Paratek Pharmaceuticals, Inc.** (Employee, Shareholder) **Diane M. Anastasiou, BA**, **Paratek Pharmaceuticals, Inc.** (Consultant)

